# A *Distributed Interactive* Decision-Making Framework for Sustainable Career Development

**DOI:** 10.3389/fpsyg.2021.790533

**Published:** 2022-02-16

**Authors:** Helen Hallpike, Gaëlle Vallée-Tourangeau, Beatrice Van der Heijden

**Affiliations:** ^1^Kingston Business School, Kingston University, Kingston upon Thames, United Kingdom; ^2^Institute for Management Research, Radboud University Nijmegen, Nijmegen, Netherlands; ^3^Faculty of Management, Open Universiteit, Heerlen, Netherlands; ^4^Department of Marketing, Innovation, and Organisation, Ghent University, Ghent, Belgium; ^5^Business School, Hubei University, Wuhan, China

**Keywords:** career decision-making, sustainable careers, distributed decision-making, interactivity, distributed agency

## Abstract

The purpose of this article is to present a new distributed interactive career decision-making framework (*di*CDM) in which person and context together determine the development of a sustainable career. We build upon recent theories from two disciplines: decision theory and career theory. Our new conceptual framework incorporates distributed stakeholders into the career decision-making process and suggests that individuals make decisions through a system of distributed agency, in which they interact with their context to make each career decision, at varying levels of participation, from proactive to reactive. We focus on two key career decision-making drivers originating from the person (exercising personal agency and seeking meaning), and two key drivers from the career context (making demands on an individual’s resources and affording scripts). This manuscript challenges the individual-driven approach to career development, and instead proposes that a process of distributed career decision-making takes place between each person and the various stakeholders, both individual and institutional, that also drive their career. Career seekers and counselors can use this framework to supplement an individual-focused approach and incorporate the role of distributed decision-makers in sustaining an individual’s career. Empirical research is needed to explore and test the applicability of the framework to career decisions in practice.

## Introduction

Developing sustainable careers in response to a changing work environment is a major challenge of the 21st century (De Vos and Van der Heijden, 2015; Lawrence et al., 2015; Van der Heijden et al., 2020), yet the complex career decision-making process which shapes a sustainable career trajectory is still not fully understood. Based on the concept of a job for life, whereby careers had predictable requirements along their entire trajectory (Wilensky, 1961), traditional models of vocational choice conceived career decision-making as a process of matching an individual’s characteristics to the work requirements (Parsons, 1909; Holland, 1959). However, from the late 20th century, academics noticed that career patterns were changing, and career frameworks began to emphasize the central role of personal agency in planning and guiding the career trajectory, suggesting that individuals had the ability to shape their own protean careers (Hall, 1996) and to move between jobs and organizations in boundaryless careers (Arthur, 1994), in a process of proactive career self-management (King, 2004; Greenhaus et al., 2019). Yet, as 21st century employment becomes increasingly volatile (DiRenzo et al., 2011; Petriglieri et al., 2019), the autonomy of individuals to control their career paths has been questioned, for example, in a re-examination of the boundaryless career (Rodrigues and Guest, 2010). Instead, building on the complementary relationship between individuals and their career context, as proposed in Super’s “life-span life-space” theory (Super, 1980). Construction theory proposes that individuals subjectively co-construct the meaning of their careers in conjunction with their career context (Savickas et al., 2009; Rudolph et al., 2019). Recent research has also emphasized the influence of the ecosystem of broad contextual factors on career choices, including society, institutions and the macro-economy (Mayrhofer et al., 2007; Baruch, 2015; Baruch and Rousseau, 2019), and has explored the consequent potential for conflict between different contextual domains, e.g., of work and family (Powell and Greenhaus, 2012; Schooreel et al., 2017). An approach to career development based on career adaptability has been advocated to enable the individual to address the changing demands of the career context (Krieshok et al., 2009; Savickas and Porfeli, 2012), and individuals have been encouraged to respond to the context by creating and grasping opportunities (Mitchell et al., 1999; Krumboltz, 2011).

The literature addressing sustainable careers emphasizes the shared responsibility of individual and context, as set out in the seminal conceptual model of De Vos et al. (2020), which forms the basis for research on sustainable careers. The authors propose that the three dimensions of a sustainable career are the *person*, whose characteristics are agency and meaning, the *context*, composed of both institutional and private stakeholders, and *time*, which introduces change and events. They suggest that the outcomes of sustainable careers are happy, healthy, and productive workers (Van der Heijden, 2005; De Vos et al., 2020), and build upon the definition of career sustainability as: “the sequence of an individual’s different career experiences, reflected through a variety of patterns of continuity over time, crossing several social spaces, and characterized by individual agency, herewith providing meaning to the individual” (De Vos and Van der Heijden, 2015, p. 7). We add to this conception of career sustainability by highlighting the interactive roles of both person and context in career decision-making and thus extending the responsibility for sustaining a person’s career to encompass individual and organizational stakeholders within the broad career context.

### Theories of Decision-Making and Sustainable Career Choice

In order to support practical career counseling, career decision-making research has often aimed to provide a normative process setting out how individuals should make career decisions, as we discuss in this section. There are different perspectives on the extent of conscious control and rational, deliberative analysis that individuals employ in making a decision. At one end of the spectrum, rational choice theories (March, 1994; Mellers et al., 1998; Powell and Greenhaus, 2012) are deductive, normative approaches to decision-making which seek to optimize decision outcomes (Chater and Oaksford, 1999; Oaksford and Chater, 2020). This approach is often applied in formal career decision-making, for example, when career seekers analyze and discuss their preferences and options with a career counselor, including applying the ‘sequential elimination’ approach in Gati’s iterative decision-making model (Gati, 1986), or applying the normative, and step-by-step process in Greenhaus et al.’s model of career management (2019). On the other hand, the limitations of theories of rational choice are addressed by alternative developments in decision theory (Fischhoff and Broomell, 2020), for example, Simon’s theory of bounded rationality (Simon, 1947) which proposes that in the face of limited data people satisfice, or make a satisfactory decision, rather than making a perfect decision; and Kahneman and Tversky’s focus on heuristics in real-life decisions which do not appear conventionally rational e.g., in their prospect theory Kahneman and Tversky (1979). These theories underpin the trilateral model of career decision-making, according to which decisions are made through a combination of intuition, reason, and engagement with the career context (Krieshok et al., 2009).

The role of interactive causality between person and context in decision-making was highlighted in Bandura’s social cognitive theory Bandura (1989), whereby individuals “make causal contribution to their own motivation and action within a system of triadic reciprocal causation, in which action, cognitive, affective, and other personal factors, and environmental events all operate as interacting determinants” (p. 1). Building on this theory, the social cognitive career theory (SCCT) (Lent and Brown, 2013, 2020) is an individual-focused, step-by-step decision-making process, which highlights the key role in decision-making played by the context in providing supports and barriers to the career, and in influencing individuals’ perceptions in the form of their self-efficacy beliefs and outcome expectations. Nevertheless, we argue that by incorporating the context in the form of external supports, barriers and influences, rather than as explicitly causal interacting determinants, the SCCT still omits part of the decision-making process.

Earlier models which focus on interactions with the career context include the dynamic interactional career development model (Vondracek et al., 1986), which highlights person-context complexity, and, subsequently, Amundson’s interactive model of career decision making [*sic*] (1995, p. 12), Amundson (1995) which explores how a decision is iteratively re-framed by the individual in response to feedback from the various “determining contexts,” which form part of the decision-making process. Current frameworks situate individual decision-making within its broader career context, addressing interactive issues such as work-family decision-making (Driver, 1980; Powell and Greenhaus, 2012; Schooreel et al., 2017), career flexibility (Tomlinson et al., 2018), the role played by agent and structure in determining an individual’s ability to move between jobs (Forrier et al., 2009), and individuals’ perceptions of career control (Guest and Rodrigues, 2015); however, all these models still understate the proactive participation of both individual and institutional stakeholders in interactive career decisions.

Each model is constructed from the perspective of the individual and not from that of the collective stakeholders in the individual’s career, and therefore individual career seekers appear more agentic and independent then they are in reality. Specifically, in the model by Powell and Greenhaus (2012), the individual makes the decision alone, whilst taking family considerations into account at each of the four decision-making stages. In addition, building explicitly upon that manuscript, Schooreel et al. (2017) analyze home-to-career interference as a potentially deterministic constraint upon, but not a proactive agent in, individual career decision-making. In a similar way, Guest and Rodrigues (2015) emphasize the influence of the context on career decisions, but they locate personal control at the center in their model of career control. Placing a greater emphasis upon the influence of the context on career trajectories, Forrier et al. (2009) focus on the interplay of agency and structure in their model of career mobility which is, however, still structured from the perspective of an individual’s decisions. Finally, Tomlinson et al. (2018) criticize the protean and boundaryless career models as overly agentic, and instead they focus on the role of organizational actors and stakeholders as career shapers which can determine the trajectory of a flexible career. Yet, ultimately they present career decisions as made by the individual.

Contextual participation in decision-making goes beyond the collective agency exercised by a group of individuals with similar intentions (Bandura, 2000), and extends to actors working together, but with potentially differing perspectives. This approach, whereby agentic social units co-operate across actors and activities (Enfield and Kockelman, 2017), can be termed distributed agency. Distributed agency is a recently developed concept which applies to agents acting in organizations and complex situations, where the actions and intentionality of multiple agents combine to produce a collective outcome. It refers to the ability of different stakeholders to participate in a single distributed process, as set out by Enfield (2017), who states that: “the elements of agency can be divided up and shared out among multiple people in relation to a single course of action” (p. 9). Distributed agency is thus not limited to one person, but shared amongst multiple stakeholders, as Enfield goes on to write (2017): “the locus of agency is the social unit, and social units are not confined to individual bodies” (p. 10). Multiple decision-making agents, who contribute to a specific decision, can be termed a decision-making unit (DMU), and are acting within a distributed decision-making process (Schneeweiss, 2003). Distributed agency thus enables decision-making to be distributed across actors and activities, for example, the distributed decision-making which takes place in situations ranging from big business problem-solving and disaster management (Schneeweiss, 2003; Treurniet and Wolbers, 2021), to doctor-patient consultations (Rapley, 2008).

We argue that there is a need to integrate the existing career decision-making models discussed above, by situating decision-making within the uncertain, but often deterministic, career context which jointly drives the process of distributed decision-making.

## A Systemic Understanding of Sustainable Career Decision-Making

De Vos et al. (2020, p. 10) have called for a “systemic perspective in which person, context, and time-related factors might dynamically interact.” We build upon their work with an interdisciplinary approach combining their research on sustainable careers with new concepts in decision-making and cognition (Secchi, 2010; Vallée-Tourangeau and Vallée-Tourangeau, 2017; Secchi and Cowley, 2021) which highlight the proactive role of the context in the decision-making process. We propose that career decisions are made jointly between individuals and the multiple stakeholders in their career context, both individual and institutional, within a system of distributed decision-making in which: “decision making [*sic*] is distributed across time, courses of actions, people, situations, and technologies” (Rapley, 2008, p. 430). The distributed career decision-making process is based upon a system of distributed agency (Enfield and Kockelman, 2017), as discussed in the section on theory above. To account for the different levels of agentic influence in any given decision outcome, we propose that the proactive participation by each participant will vary from one career decision to the next, and that the level of participation of each decision-maker will be determined both by objective structures, such as organizational hierarchy, and subjective motivations, for example, the level of engagement of the various distributed decision-makers with the specific decision.

### A New *Distributed Interactive* Career Decision-Making Framework for Sustainable Careers

**FIGURE 1:**
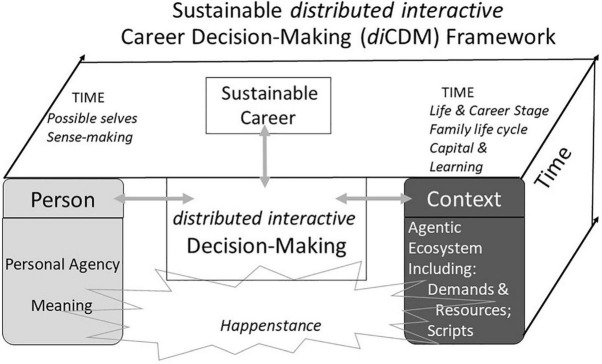
A Sustainable *distributed interactive* career decision-making framework.

Our new framework introduces the concept of distributed decision-making to career decisions to explain how both person and context interact with intentionality in making sustainable career decisions. The *di*CDM framework presented here (see Figure 1) focuses on the multiple agents taking part in the career decision-making process. Supplementary Appendix 1 illustrates how our framework can be applied to career decisions over the course of a fictional career.

### Person

The person is the first dimension of a sustainable career identified by De Vos et al. (2020), and we build upon their identification of agency and meaning as two key personal career drivers.

#### Exercising Agency

Personal agency is exercised by individuals when they “intentionally make things happen” (Bandura, 2001, p. 1). Individuals exercise agency in the process of career self-management (King, 2004) and goal setting (Latham and Locke, 1991). An individual employs their agency to fulfill a range of psychological needs, which consequently become salient in their career choices, including the need for self-determination and connection to others (Duffy et al., 2016; Ryan and Deci, 2017) (see below under Meaning). Agency is also influenced by the individual’s expectations, for example, by their perceptions of self-efficacy (Betz and Hackett, 1986; Bandura, 2001; Lent and Brown, 2013); individual character traits such as resilience and adaptability (Bimrose and Hearne, 2012) also support an individual’s readiness to exercise their personal agency. Inaction in careers (Verbruggen and De Vos, 2020) can also be explored from the perspective of the presence or absence of agentic decision-making. A useful starting-point to operationalize personal agency in career decision-making could be the definition and measurement of career control (Guest and Rodrigues, 2015), combined with objective facilitating factors such as accumulated career capital, defined as the development, over time, of key career competencies, which have been identified by Arthur et al. (1995) as work motivation (*knowing why)*, skills (*knowing how*), and networks (*knowing whom*). Collective agency (Bandura, 2000) denotes when a group of people work together to achieve a goal. Enfield and Kockelman (2017) take the concept of agency one step further, and propose that “distributed agency” is shared between multiple stakeholders in agentic social units. We develop this perspective in the section on context below.

#### Seeking Meaning

Sustainable careers have been described as linking individual agency and personal meaning (Van der Heijden and De Vos, 2015). Meaningful work has been widely discussed and operationalized in the career literature. In particular, people are motivated to work to gain the intrinsic rewards of pursuing a broader life purpose, and work can be a way in which people find or actively create meaning and fulfillment in their lives (Steger et al., 2012). They often derive multiple social and other benefits from the work environment, which address their need for relatedness (Ryan and Deci, 2017) and social connection (Duffy et al., 2016), to answer their basic psychological needs (Allan et al., 2019); indeed, to fulfill a quest for meaningful work (Bailey et al., 2019), they may choose meaning in preference to higher salaries (Hu and Hirsh, 2017). A career can afford meaning and purpose by providing objectives, such as the pursuit of ambitions (Judge and Kammeyer-Mueller, 2012). Meaning in a career may be created in a process of retrospective sense-making (Weick, 1995; Wrzesniewski et al., 2003), which in turn feeds forward into future career decisions, and job crafting (Wrzesniewski et al., 2013), or meaning may be co-constructed through social discourse (Savickas et al., 2009; Del Corso and Rehfuss, 2011). Sense-making also informs career narratives in career research as participants may selectively omit inconsistencies to construct a coherent narrative (Wolf, 2019). A particular form of meaningful work to which the individual is highly committed is termed a ‘calling’ (Hall and Chandler, 2005; Berg et al., 2010; Lysova et al., 2019), often described as a response to an external “transcendent summons” (Dik and Duffy, 2009, p. 427), a description which provides further evidence to explore the interactivity between person and context. However, a calling can be a double-edged sword (Lysova et al., 2018), since a strong commitment to personal professional development can enhance employability, but if the person’s commitment to their calling reduces their flexibility regarding their work role, this can be detrimental to their career sustainability.

Agency and meaning have thus been identified as two key components of a person’s decision-making to sustain their career. We add to this identification of individual agency and argue that the person makes decisions in conjunction with a distributed network of active agentic decision-makers within the wide career context, to which we now turn.

### Context

The context is the second dimension of a sustainable career as identified by De Vos et al. (2020), and comprises the multiple stakeholders in an ecosystem of political, economic, work, and social structures (Baruch, 2015; Baruch and Rousseau, 2019; Gribling and Duberley, 2020) which form supports or barriers to an individual’s career development (Lent and Brown, 2013, 2020). We conceptualize the context, as well as the person, from an agentic perspective, that is, we attribute intentionality to various agents in the broader career ecosystem, who therefore contribute proactively to each career decision. The career ecosystem is thus an external driver of individuals’ career decision-making. Not only does it encompass broad distal influences, such as political and legal systems, but also, more specifically, it includes proximal influences, which play an active role in decision-making. For example, stakeholders from family and work, who make demands upon individuals’ resources, and socially created systems and structures, or for instance, career paths, which offer career scripts to individuals. We thus focus specifically on two key characteristics of the career context which drive career decisions: making demands on an individual’s resources, and affording scripts. These characteristics represent two key aspects of the context: the embodied demands of other individuals e.g., family members or one’s boss, and the abstract collective scripts which can determine individuals’ decisions in their public and private lives.

#### Making Demands on Resources

A sustainable career enables individuals to continue to earn their living over time and to benefit from social interaction, fulfilling the basic needs of competence, autonomy and relatedness (Ryan and Deci, 2000). Personal relationships and circumstances (including family and financial needs) place practical external demands and constraints on individuals’ financial resources, including to provide for one’s own and others’ survival needs (Duffy et al., 2016). The work-family literature highlights that the career environment also places demands upon individuals’ time and energy, and these demands can result in career choices driven by contextual demands rather than by individual preferences (Powell and Greenhaus, 2012). Individuals are motivated to protect and build their resources, for example, their energy, their self-esteem, and their social status, in order to enhance the positive reinforcement that they experience from their environment, and to avoid stress caused by the loss of these resources, according to the Conservation of Resources theory (COR) (Hobfoll, 1989). If demands and resources are not balanced over time, i.e., if they do not maintain a dynamic balance, this can give rise to stress and burnout as explained by the “job demands-resources” model of burnout (JD-R) (Demerouti et al., 2001), which proposes that if the physical and emotional demands made by a job are too great, this leads to exhaustion, and if the resources offered by the work are inadequate, this leads to disengagement; each effect can moderate the other, and in both cases this would lead to making their career unsustainable.

#### Affording Scripts

Scripts are schemata shared by a group of people which guide their behavior over the course of a standardized series of events (Schank and Abelson, 1977; Gioia and Poole, 1984). Career scripts are a specific form of script which structure expectations about the development of careers (Dany et al., 2011); they were recently operationalized as a template that workers can follow: “collectively shared interpretive schemes that describe successful careers” (Laudel et al., 2018, p. 932). Scripts can be acquired by selecting role models through observing the career trajectories of reference groups (Grote and Hall, 2013). We suggest that scripts are not static but develop over time as individuals collectively and proactively interact with the career context, thus creating new scripts, in the same way as individuals co-construct their own career trajectories through discourse with, and activity within, the context (Savickas et al., 2009, p. 246): “Each person constructs reality through verbal discourse yet what they do is a major component in the evolution of this discourse.” Career scripts are constructed and enacted by individuals in organizations, collectively creating an internal labor market and writing career development plans, and we further suggest that the concept of scripts can extend to incorporate the contextual influence of family values, expectations and aspirations which inform career orientations (Rodrigues et al., 2013) and that therefore career orientations, and the earlier concept of career anchors (Schein, 1996), can be considered an external, as well as internal, source of motivation. Scripts therefore reflect the collective agentic interests of multiple stakeholders, such as institutions, which use career scripts to structure their internal labor markets and motivate current and future employees, or family and friends, for whom scripts may embody lifestyle values and aspirations.

### Time

Time is the third key dimension of a sustainable career identified by De Vos et al. (2020). To reflect this, our *di*CDM framework incorporates life and career stages which have been associated with individual changes over time. These may be based on chronological age (Super, 1957; Levinson, 1986) but may also reflect a pattern of behaviors as an individual recycles through the family life cycle in a second marriage, or through career stages on changing jobs (Super, 1990; Smart and Peterson, 1997). Objectively, the passage of time can increase career opportunities, as past experiences feed forward through time, building up individuals’ learning and human capital, with the result that employability increases over time, with experience or transferable skills (Fugate et al., 2004). Subjectively, over time, in a process of sense-making, individuals create coherent past career narratives to underpin their identity and create meaning in their work, and in turn, these may influence their future decisions (Del Corso and Rehfuss, 2011; Wrzesniewski et al., 2013) as well as their dreams and fears of future possible selves (Markus and Nurius, 1986).

#### Affording Happenstance

Happenstance emerges naturally and chaotically over time from a dynamic environment (Bright and Pryor, 2005; Bright et al., 2005). Over time, chance events, over which the individual has no control, can be core influences on individual lives (Bandura, 1982; Grimland et al., 2012; Kindsiko and Baruch, 2019) and individuals have been encouraged to learn to recognize and exploit them to optimize their career success (Krumboltz, 2009, 2011). Akkermans et al. (2018) discuss how individual agency interacts reciprocally with contextual factors in the form of career shocks, which can be positive or negative, and suggest that existing career decision theories such as career construction theory (Savickas et al., 2009) and social cognitive career theory (Lent and Brown, 2013, 2020) are still overly agentic and goal-directed. Recent work highlights the effect of early positive and negative career shocks on career sustainability (Blokker et al., 2019) and Pak et al. (2020), found that organizations can assist in mitigating the impact of career shocks to assist with career sustainability.

### Interactivity and Distributed Decision-Making

The 21st century workers are neither in control of their careers, nor are they passive laborers: they are negotiators, interactively developing their own career trajectories in a shifting multilateral relationship with stakeholders with different agendas, who may be collaborative, antagonistic, or simply demanding, and who occupy different positions within different contextual structural hierarchies. We place distributed interactivity at the center of our career decision-making framework, and, building on concepts of distributed cognition and decision-making (Secchi, 2010; Vallée-Tourangeau and Vallée-Tourangeau, 2017; Secchi and Cowley, 2021), we propose that career decision-making results from the interaction between the person and the distributed drivers in their career context. Our focus on distribution and interactivity as essential mechanisms in the decision-making process therefore addresses the question as to the extent to which individuals perceive that they exercise, or are perceived to exercise, agency in their own career decisions.

In order to assess and operationalize the level of participation in each career decision, the *di*CDM framework proposes that each distributed decision-maker participates at a level which can be envisaged on a continuum of interactive decision-making participation; this continuum ranges from primarily proactive decisions, whereby the decision-maker exercises almost complete autonomy, to primarily reactive decisions in response to other stakeholders in the career context. We therefore propose that for each decision, each agent in the network of distributed decision-makers will have greater or lesser input into the outcome, which could be operationalized as ranging from 0 to 1, in other words from no agentic input to complete decision-making autonomy; for measurement purposes this could be based on levels of input reported by the stakeholder and triangulated by the other participants in the decision.

#### Proactive Participation

Proactive personality has been described as a stable trait of a person who takes the initiative and creates positive change in his or her environment, relatively unconstrained by their situation (Seibert et al., 2001). We suggest that proactive behavior may be exercised by any agent, at any point along the career trajectory in the career decision-making process, depending on their circumstances and engagement with the particular decision. Proactive decision-makers exercise agency in implementing plans to pursue meaning by thoughtfully researching and selecting their role and employer, or by job crafting (Plomp et al., 2016), and they manage their careers, for instance, by networking to create social capital to provide career opportunities (King, 2004; Jacobs et al., 2019). Individuals can even proactively manage their career timetables, for example, by studying for qualifications to bring forward a promotion, or later they may proactively choose their retirement date, as their priorities change, for example, when they become grandparents (Bailyn, 2004). Through these activities, individuals proactively restructure their career context, and, meanwhile, multiple stakeholders make proactive decisions which change the career context, and these intentionally or indirectly alter the trajectory of an individual’s career. Last but not least, career inaction can also be a deliberative choice: the recognition of career desires may influence an individual to continue in the same role (Verbruggen and De Vos, 2020). We suggest that proactively planned career choices may enhance or, alternatively, detract from an individual’s career success and sustainability, and they may not be inherently either more or less beneficial than the reactive choices discussed below.

Perceptions of proactivity can be operationalized both as the control that individuals and stakeholders report that they exercise over their careers, but also by the career-enhancing behaviors in which they proactively participate, such as networking by the individual or the decision by the employer to provide training.

#### Reactive Participation

Reactive decision-making responds to the context and this approach leads the individual to acquiesce to the demands made by people and organizations in their wider career context and to accept contextual scripts, norms and expectations. Ultimately, individuals have no choice in some decisions which determine their career trajectory, such as redundancy, which are imposed on them by an external hierarchy, such as that of their employer. Adolescents’ career aspirations may not be formed as deliberate choices but instead, they can be circumscribed by their passive reaction to prevailing social norms (Gottfredson, 2005). Individuals may choose to adopt the roles suggested by their environment, for instance, when they follow an expected script and choose a career to fit with family or ethnic group values and expectations (Lee et al., 2011; Rodrigues et al., 2013), and this reactive approach may or may not be beneficial to them. Furthermore, individuals react over time to changes in the economic, political, and technological context (Lone et al., 2015), and to the social norms governing the length of episodes of employment (Petriglieri et al., 2019). Individuals may be reactive because they do not have clarity about their career goals or paths, instead passing through a mist of indecision (Suzuki and Kato, 2006). In certain circumstances, passive acceptance of the current norms can be disadvantageous, for example, if workers do not actively update their skills, they may be rendered obsolete as technology reconfigures job content and therefore job requirements (Frey and Osborne, 2017). Institutions and family can also be reactive in their decision-making, acquiescing to a proactive individual’s decisions and actions. We argue for the need to redefine the context as an active driving force for career decisions within a system of distributed career decision-making, such that when the person is reactive there is, with the exception of chance events arising through happenstance, usually a corresponding active decision-maker within the broader career context, for example, a colleague, a family member, or a more abstract agentic force, such as family expectations or institutional demands. In addition, most decision-making will be multilaterally interactive and distributed between stakeholders, as discussed below.

#### Distributed Participation

Whilst we employ the concepts of proactive and reactive to identify the two ends of the continuum, most decision-making is distributed and interactive. Interactive decision-making is reciprocal and iterative, so interactive decision-makers both shape, and are shaped by, their career context. Individuals’ career decisions cumulatively construct their career trajectories, and these, in turn, confirm or re-write the career scripts which shape their career context, and in this way, individuals enact their own careers and produce part of the context with which they themselves then continue to interact (Weick, 1995; Gottfredson, 2005). In a similar vein, career construction theory (Savickas et al., 2009) proposes that the meaning of a career is co-constructed by the individual and the environment. However, interactive career decision-making is more than the subjective co-construction of meaning: it is also the objective co-construction of career trajectories themselves by multiple stakeholders in a system of distributed decision-making, whereby key decisions about an individual’s career are made not only by the individual but multilaterally by agentic decision-makers or by chance events. To sustain a career in a dynamic workplace, career adaptability is an essential interactive skill (Fugate et al., 2004; Savickas and Porfeli, 2012). Parents may adapt their careers to fit around the demands of their families (Mainiero and Sullivan, 2005), and dual career couples may interactively adapt to sustain their own and their partner’s career (Petriglieri and Obodaru, 2018). To sustain their careers, workers will need to adopt an interactive approach to the career context and grasp opportunities which arise (Mitchell et al., 1999; Krumboltz, 2011).

## Discussion

This manuscript presents a new *distributed interactive* career decision-making framework (*di*CDM) based on an interdisciplinary perspective on career sustainability, which applies recent developments in decision theory and theories of career sustainability to understand the mechanisms underlying sustainable career decision-making. We have discussed the key approaches to decision-making in the current literature, including the latest research on distributed agency and distributed decision-making theory, which we have applied to career decision-making. We have also reflected on the extant literature on sustainable careers and career decision-making, and we build upon recent research into the contextual influences on careers to argue that current models of career decision-making continue to be overly agentic, in that they present the individual as the sole decision-maker driving the career trajectory. We argue that, whilst contextual influences and happenstance have been researched in the recent literature, they have hitherto been included in the career decision-making models as external influences, affordances, constraints, or barriers to the individual decision-maker. In other words, there has been no unifying model which integrates the input of other members of the DMU, ranging from institutions and organizations to family members, into individual career decisions. In contrast, our model integrates the contextual stakeholders into the decision-making process as members of the DMU, who participate as multiple agents in a system of distributed decision-making. Our model also addresses the level of active input, by each stakeholder, into any given decision, along a continuum ranging from proactive to reactive participation, and thus we incorporate the option of each stakeholder to exercise their agency to a greater or lesser degree within the decision-making process.

### Theoretical Implications

We have set out how our systemic approach to career development differs from the individual-focused approach in the career literature, according to which the key career driver is the person, and the role of the context is to provide supports and barriers to the chosen career trajectory. We build instead upon recent work on the shared responsibility between person and context emphasized in the sustainable career literature. Moreover, we have argued how our proposed process differs from the classical decision-making approach, and instead builds on recent concepts of distributed cognition and systemic thinking (Secchi, 2010; Vallée-Tourangeau and Vallée-Tourangeau, 2017; Secchi and Cowley, 2021), distributed agency (Enfield and Kockelman, 2017), and distributed decision-making (Schneeweiss, 2003; Treurniet and Wolbers, 2021). To account for the role of the diverse agents, we propose that their level of participation in each career decision varies along a continuum from proactive to reactive, as circumstances vary. We suggest that the primary added value of this manuscript is to make a contribution to theory (Colquitt and Zapata-Phelan, 2007) by applying recent concepts from the decision-making literature to provide a new distributed interactive understanding of career decision-making, and to integrate the phenomenon of decision-making into the sustainable career literature.

### Practical Implications

Traditional career development advice has been criticized for its focus on those aspects of individuals’ careers that they can proactively control (Mitchell et al., 1999). Career advisors need to recognize that careers are less under rational individual control than the literature traditionally suggested (Arthur, 1994; Arthur et al., 1995; Hall, 1996), and instead to advise individuals to interact imaginatively with an envisaged possible career (Markus and Nurius, 1986; Loveday et al., 2018). Instead of job matching (Parsons, 1909; Holland, 1959), which in a rapidly changing work context may be only short-term, or making superficially rational choices between pre-determined longer-term career scripts (Laudel et al., 2018), career counselors need to emphasize that individuals and their career context are interdependent, and that neither is static, but that they each change through time and through their reciprocal interaction. A counselor who seeks to provide support for a sustainable career needs to be able to help the individual to think through the different driving forces in their lives at any given point, to recognize affordances, that is, action possibilities, in their career context (Krumboltz, 2011), and to reflect on where and when it is important (or even possible) to drive the decision-making process, and when it is advantageous to allow it to be driven by the agentic decision-makers in the career context. Finally, as we highlight the role of stakeholders in the wider career context (Baruch and Rousseau, 2019), we also highlight the responsibility of macroeconomic decision-makers such as governments and organizational decision-makers such as managers, to act as proactive stakeholders to support sustainable careers, for example through legislation, and to reduce constraints, for instance, by means of flexible HR practices.

### Future Research

As this is a new conceptualization of career decision-making, it first needs to be explored qualitatively, for instance through individual interviews, to ascertain its relevance to different types of individual career and career decisions. Research needs to operationalize the concept of distributed decision-making in such a way that the inputs of the range of potential decision-makers can be captured, so a wide range of stakeholders DMU in a decision (Schneeweiss, 2003), can be interviewed, to triangulate the results and compare whether all participants have the same perspective about who made each decision. After initial qualitative exploration, larger-scale quantitative surveys could explore the level of control that individuals reported that they exercised in a recent key career decision, building on research by Guest and Rodrigues (2015). Research would be useful into the decision-making styles of workers in careers with high turnover and low sustainability in the gig economy (Petriglieri et al., 2019), such as taxi drivers, and also in embodied careers of sportspeople, entertainers e.g., ballet dancers, or the armed forces, that is, in jobs which are not designed to last a full lifetime (Rodrigues et al., 2020), to research what constitutes sustainable decision-making in those circumstances and to identify the various decision-making stakeholders. To check generalizability, it would be useful to explore a wide socio-demographic range of participants, for example, older workers and millennials; workers at different family life cycle stages, including second families; and respondents from deprived social groups. Sustainable careers and decision-making could also be researched in different employment conditions, such as amongst low-paid workers, entrepreneurs and gig economy workers. A key question to explore for career development today is: to what extent is it possible to develop sustainable careers proactively, employing, for example, the agentic approach of the intelligent career (Arthur et al., 1995)? And in what circumstances is it optimal to manage one’s career proactively, to follow the organizational script reactively, or to engage interactively? Questions could be asked regarding decisions when workers exercised different levels of control and engagement (Guest and Rodrigues, 2015).

It would be useful to explore whether any common factors can be identified in relation to career sustainability in terms of personality, circumstances or proactive or reactive decision-making styles, so personality tests could be used to ascertain which participants were naturally more proactive or control-seeking (Seibert et al., 2001), and whether this was a factor in their decision-making and their career sustainability. Ideally, situations would be explored where participants perceived that they made decisions “in and out of character” to see which situational factors might be related to certain career decision-making behaviors. It would be useful to find out if there is any relationship between perceived success in careers, objective or subjective, Grimland et al. (2012) on the one hand, and individuals’ reported level of proactivity in their career decisions, on the other hand (Seibert et al., 2001). The extent to which stakeholder participation in decision-making can affect the success of career outcomes can be researched from an interactivity perspective, building on organizational studies and work-family literature. Different age groups could be studied simultaneously or one group could be studied longitudinally using a person-centered research method (Laursen and Hoff, 2006; Morin et al., 2018) to analyze the extent to which individuals express concerns about career and income sustainability and alter their decision-making priorities and proactivity in career decision-making in different circumstances and at different career stages. It would be useful to see whether the level of an individual’s work or career centrality (Hirschfeld and Feild, 2000) affects their career choices and whether this remains consistent over time or changes with personal circumstances, and if so, how this related to career adaptation (Fugate et al., 2004). Finally, in the current hybrid working conditions introduced by the Covid-19 pandemic, our model could be helpful in exploring changes in decision-making during times of increased proactive involvement of government in individual and organizational decision-making. In particular, it could serve as an example of a previously distal influence becoming a proactive stakeholder in decisions such as whether to work from home, and even whether a sector of the economy (e.g., travel or hospitality) is viable. Furthermore, research has already found that women have taken on greater childcare responsibilities during the pandemic (Clark et al., 2021), and our model could therefore be used to investigate this phenomenon to explore the development of decision-making processes within family households throughout the pandemic, and the extent to which each household member perceives their ability to make proactive decisions about home-working and childcare. It would also be useful to explore whether the altered working conditions have changed perceptions and attitudes for the future in an envisaged post-pandemic labor market.

### Conclusion

This manuscript contributes to our understanding of sustainable career decision-making from a distributed interactive perspective. We propose a distributed interactive career decision-making framework (*di*CDM) in which the career decision-making process is a system of agentic interactions, both individual and collective, between individuals and multiple stakeholders in their career context, that repeats interactively through time. The *di*CDM framework sets out how personal drivers (exercising agency and seeking meaning) interact reciprocally with contextual drivers (making demands on resources and affording scripts), all of which are influenced by happenstance, as chance events arise over time. We thus argue that career decisions are made, not by one individual alone, but through the interaction between person and career context over time, in a system of distributed agency. This distributed agency in turn enables distributed decision-making, whereby the person and contextual stakeholders interact multilaterally to participate in each decision to a greater or lesser degree. Our focus on distributed decision-making interactivity contributes to the concept of shared influence over, and therefore shared accountability for, sustainable careers. We further suggest that the level of participation in each decision by any agent will vary from one decision to the next, depending upon their circumstances and level of subjective engagement, along a continuum from proactive to reactive participation, and we discuss how this might be measured. We have suggested possible applications of our framework and made recommendations for future research.

## Author Contributions

HH proposed the original study and wrote the first draft of the manuscript. HH, GV-T, and BV contributed to the development of the concepts, suggestions, and amendments to the manuscript. All authors contributed to manuscript revision, read, and approved the submitted version.

## Conflict of Interest

The authors declare that the research was conducted in the absence of any commercial or financial relationships that could be construed as a potential conflict of interest.

## Publisher’s Note

All claims expressed in this article are solely those of the authors and do not necessarily represent those of their affiliated organizations, or those of the publisher, the editors and the reviewers. Any product that may be evaluated in this article, or claim that may be made by its manufacturer, is not guaranteed or endorsed by the publisher.
