# Supplementary material for: A Distributed Interactive Decision-Making Framework for Sustainable Career Development
Source: Front Psychol. 2022 Feb 16;12:790533. doi: 10.3389/fpsyg.2021.790533 (PMC8896176; doi:10.3389/fpsyg.2021.790533)
Supplement: Supplementary file 1 [file Table_1.DOCX]

***Fictional case illustrating person interacting with agentic career context (Appendix 1)***

Anna’s family wanted her to join the family business on leaving university (script), but she insisted on gaining professional qualifications and experience (proactive personal agency, capital) to ensure that her career was sustainable (sustainable career), so she completed training as a management accountant (career script). She worked in a large firm in London but once she had her first child (family life cycle), who needed her time and energy (demands and resources), commuting to London became more difficult. Her boss supported flexible working (proactive context) so she negotiated (interactive) to work shorter hours to be able to continue in her role (sustainable career). Then came an economic recession (ecosystem) and her employer made her redundant (happenstance) and to keep his job her husband needed to work away from home in the week (sustainable career). She took the opportunity to stay home with her children for a couple of years, which she found fulfilling (meaning). However, money was then running short (demands and resources), and when the children went to school, she set up a partnership to provide local accounting services (personal agency), in response to an initiative by a government minister encouraging female entrepreneurs (proactive context). Not only did this job provide income (demands and resources) and fit quite well round her family, but she also enjoyed her work and felt a sense of achievement and purpose (meaning).
